# Effect of Graded Levels of Fenugreek (*Trigonella foenum-graecum* L.) Seeds on the Growth Performance, Hematological Parameters, and Intestinal Histomorphology of Broiler Chickens

**DOI:** 10.3390/vetsci9050207

**Published:** 2022-04-23

**Authors:** Deependra Paneru, Guillermo Tellez-Isaias, Nicholas Romano, Gautami Lohakare, Walter G. Bottje, Jayant Lohakare

**Affiliations:** 1 Department of Agriculture, University of Arkansas at Pine Bluff, Pine Bluff, AR 71601, USA; panerud9890@uapb.edu; 2 Center of Excellence in Poultry Science, University of Arkansas, Fayetteville, AR 72701, USA; gtellez@uark.edu (G.T.-I.); wbottje@uark.edu (W.G.B.); 3 Department of Aquaculture and Fisheries, University of Arkansas at Pine Bluff, Pine Bluff, AR 71601, USA; romanon@uapb.edu; 4 Little Rock Central High School, 1500 S Park St, Little Rock, AR 72202, USA; 1705shankul@gmail.com

**Keywords:** phytogenic feed additives, fenugreek, broilers, growth performance, intestinal histomorphology

## Abstract

Two experiments were conducted to evaluate the effects of fenugreek seeds (FS) as a potential alternative to antibiotic growth promoters in broiler chickens. In the first experiment, one-day-old Ross (n = 160) straight-run broilers were fed FS at 0 g, 2.5 g, 5 g, and 10 g/kg of diet during the starter (from 1 to 21 days) and finisher phase (from 22 to 35 days) with four replicates of ten birds each. In the second experiment, one-day-old Ross (n = 144) male broilers were fed 0 g, 5 g, and 10 g FS per kilogram of diet during the starter (from 1 to 21 days) and finisher phase (from 22 to 42 days) with six replicates of eight birds each. In addition to growth performance, hematological parameters and intestinal histomorphology were measured in the second experiment. FS linearly reduced the body weight gain (BWG) (*p* < 0.001), feed intake (FI) (*p* < 0.05), and increased feed conversion ratio (FCR) (*p* < 0.05) during the starter phase in both experiments. However, no significant effects on BWG, FI, and FCR were observed during the finisher phase. Moreover, the overall BWG and FI were linearly reduced (*p* < 0.05) with the increasing levels of FS, but BWG and FI were similar in the 5 g/kg FS group and control group. The inclusion of FS had a linear increase in white blood cell (WBC), heterophil, and lymphocyte count (*p* < 0.005) and the decrease in hematocrit % (*p* = 0.004) and total bilirubin (*p* = 0.001). The villus height and villus height: crypt depth ratio of jejunum and ileum were significantly lower in 5 g FS and 10 g FS treatments (*p* < 0.001) compared to the control. The result indicates that the dietary inclusion of FS reduces the early growth performance, increases the WBC counts, and negatively affects the intestinal morphology of broiler chickens.

## 1. Introduction

Antibiotics are used in poultry production to promote rapid growth and disease prevention [1]. For more than 60 years, antibiotics have been supplemented in animal feed at subtherapeutic doses to improve growth and feed conversion efficiency and prevent infections [2]. However, the antibiotics used in poultry feed have increased the health concerns in humans due to the production of resistant strains of pathogenic bacteria [3] and the bioaccumulation of antibiotic residues in poultry meat and eggs [4,5]. The human intake of poultry products containing residues above safe maximum residual levels is associated with numerous health hazards such as allergic reactions, dermatitis, alteration of intestinal microflora, development of antibiotic resistance, and sometimes drug toxicity [6,7]. In response to health concerns, food regulatory and health authorities have restricted antibiotic growth promoters (AGPs) in poultry feed, which has intensified the search for a non-antibiotic alternative in poultry feed to maintain or improve poultry health and performance [8]. Prebiotics, probiotics, synbiotics, and phytogenic feed additives (PFA) have been presented as potential alternatives to AGPs [9]. The demand for phytogenic feed additives has increased due to the growing preference for natural and safe alternatives, enabling them to be utilized in poultry feed to improve productivity and replace AGPs.

The PFA used in the present study is the fenugreek (*Trigonella foenum-graecum* L.) seeds powder, which has been reported to possess hypoglycemic [10], hypocholesterolemic [11], antioxidant [12], immunomodulatory [13], digestion enhancing, anthelmintic, antibacterial, anti-inflammatory, antipyretic, and antimicrobial effects [14]. Fenugreek seed contains bioactive constituents such as alkaloids, steroids, flavonoids, and saponins [15]. Our previous study with the dietary inclusion of 15 g/kg of FS and a blend of probiotics also improved the growth performance and nutrient digestibility of New Zealand White rabbits [16]. Similarly, studies with the dietary inclusion of 5, 10, and 15 g/kg FS [17], 10 g/kg normal, and enzyme-treated FS [18] reported improved growth performance in broilers. In contrast, other studies have reported a reduction in growth performance of broiler with the inclusion of 5 g/kg FS at 42 days of age [19], 5 and 10 g/kg FS at 21 days of age [20], 30 g/kg FS at 21 and 28 days of age [21], and 40 and 50 g/kg FS at 38 days of age [22]. However, the limited studies on broilers do not provide enough evidence to support the hypothesis that the inclusion of FS may improve the growth performance and overall health of broiler chickens.

This study aims to determine the effect of different levels of fenugreek seeds on the growth performance, hematological parameters, and intestinal histomorphology of straight-run and male broiler chickens. We hypothesized that the overall performance and intestinal histomorphology of the broilers would be positively affected by fenugreek seeds consumption.

## 2. Materials and Methods

### 2.1. Preparation of Fenugreek Seeds

Dried fenugreek seeds (Deep Foods Inc., Union City, NJ, USA) were obtained from the local vendor in Little Rock, AR. Seeds were ground in a grinding grain mill (Thomas Scientific, Swedesboro, NJ, USA) using a 1 mm sieve to bring them to medium texture. Ground seeds were evenly mixed with the starter and finisher diets as per the requirements in the experimental designs of the two experiments. The analysis of fenugreek seeds was conducted in our previous study by Abdel-Wareth et al., 2021, using gas chromatography–mass spectrometry [16].

### 2.2. Animals and Experimental Design

In the first experiment, one-day-old straight-run broiler chicks (Ross, n = 160) were weighed on arrival from the hatchery (Keith Smith, Hot Springs, AR, USA) and randomly allocated to 16 floor pens (10 birds/pen). Each floor pen (71 × 35 × 19 inches) was covered with pine wood shavings and equipped with a separate feeder (Harris Farms Hanging Feeders, Tractor Supply Co., Pine Bluff, AR, USA) and drinker (Harris Farms Drinkers, Tractor Supply Co., Pine Bluff, AR, USA). Four pens were randomly assigned to 1 of 4 treatments in a completely randomized design. The dietary treatments included a corn-soybean-based basal diet with the inclusion of FS powder at 0 g, 2.5 g, 5 g, and 10 g per kilogram of the diet. The basal diets (Table 1) were formulated for starter (from 1 to 21 days) and finisher period (from 22 to 35 days) to meet or exceed the nutrient requirement of NRC, 1994.

In the second experiment, one-day-old male broiler chicks (Ross, n = 144) were weighed on arrival from the hatchery (Keith Smith, Hot Springs, AR, USA) and randomly allocated to 18 floor pens (8 birds/pen). Each floor pen was covered with pine wood shavings and equipped with a separate feeder (Harris Farms Hanging Feeders, Tractor Supply Co., Pine Bluff, AR, USA) and drinker (Harris Farms Drinkers, Tractor Supply Co., Pine Bluff, AR, USA). Six pens were randomly assigned to 1 of 3 treatments in a completely randomized design. The dietary treatments included a corn-soybean-based basal diet with the inclusion of 0 g, 5 g, and 10 g FS powder per kilogram of diet. The basal diets (Table 1) were formulated for starter (from 0 to 21 d) and finisher period (from 21 to 42 d) to meet or exceed the nutrient requirement of NRC, 1994.

In both experiments, the feed was provided in mash form. All the birds were offered ad libitum access to feed and water during the experiments. The ambient temperature was gradually decreased from 34 °C (40 RH%) for days 1–3, 31 °C (40 RH%) for days 4–6, 29 °C (35 RH%) for days 7–10, 27 °C (45 RH%) for days 11–14, and 25 °C (35 RH%) thereafter.

### 2.3. Sample and Data Collection

Birds were weighed on a pen basis after arrival from the hatchery (d 1), at the end of the starter period (d 21), and at the end of the finisher period (d 35 in the first experiment and d 42 in the second experiment) to calculate mean BW and BWG. Feed consumption was recorded on a pen basis for the starter and finisher period to calculate FI and FCR. For the second experiment, two birds were selected from each pen on d 42 that represented the pen, individually weighed, and euthanized by decapitation technique. Blood was collected from the jugular vein in two different tubes for whole blood and serum collections.

### 2.4. Hematological Parameters

On day 42, blood samples were collected from a jugular vein of two birds per pen into 5 mL BD Vacutainer EDTA blood collection tubes (Becton, Dickinson and Company, Franklin Lakes, NJ, USA) to collect whole blood. Whole blood samples were analyzed for white blood cell (WBC) count, hemoglobin, hematocrit, total protein, heterophils, lymphocyte, monocyte, and basophil counts. Another 5 mL of blood was collected in a BD Vacutainer serum tube (Becton, Dickinson and Company, Franklin Lakes, NJ, USA) to obtain blood serum. The blood was allowed to clot for approximately one hour at room temperature and then centrifuged at 2000× *g* for 15 min in a Centrifuge 5430R (Eppendorf SE, Enfield, CT, USA) to collect the blood serum. Serum samples were analyzed for glucose (GL), blood urea nitrogen (BUN), albumin (A), globulin (G), albumin/globulin ratio (A: G), total bilirubin (TB), and gamma-glutamyl transferase (GGT). Whole blood and serum samples were analyzed by an external laboratory (Arkansas State Veterinary Laboratory, Little Rock, AR, USA).

### 2.5. Intestinal Histomorphology

Intestine samples (about 2 cm segments) were collected from the mid-region of the intestinal segments: jejunum (from the pancreatic loop to Meckel’s diverticulum) and ileum (from Meckel’s diverticulum to the ileocecal junction) of two birds per pen. The samples were washed with 0.1 M phosphate-buffered saline (pH 7.4) and then fixed in Bouin’s solution for 24 h, followed by preservation in 70% (*v/v*) ethanol for a day. The samples were then progressively dehydrated at increasing concentrations of ethanol, which was then cleared in xylene. The samples were then embedded with paraffin wax and sectioned at 5 µm thickness using Manual Rotary Microtome (Leica Biosystems, Buffalo Grove, IL, USA). Two sections, at different depths, were made for each sample, which was then stained with hematoxylin and eosin. Pictures at ×50 magnification were taken, and the villus height, width, and crypt depth were measured from five random villi from each replicate using Leica software (Leica DM3000, Leica Biosystems, Buffalo Grove, IL, USA).

### 2.6. Statistical Analysis

Replicate pens were considered as the experimental unit for all analyses. Data collected for growth performance, hematological parameters, serum biochemistry, and intestinal histomorphology were analyzed by one-way analysis of variance (ANOVA) using IBM SPSS Statistics V28.0 software (IBM Corp., Armonk, NY, USA), evaluating the effect of dietary FS inclusion levels by polynomial contrasts. Duncan’s multiple range test was used to compare the difference among the group means. *P*-values ≤ 0.05 were considered statistically significant, and 0.05 ≤ *p*-values ≤ 0.10 were considered a tendency. Results were presented as means and their pooled standard error.

## 3. Results

### 3.1. Feed Analysis

The feed analysis of the experimental basal diet is summarized in Table 2. The starter and finisher basal diet contained 92.3% dry matter and 23.1% and 18.9% crude protein, respectively.

### 3.2. Major Constituents of Fenugreek Seeds

The extract of fenugreek seeds powder contained following major active components as analyzed in our previous study by Abdel-Wareth et al., 2021 [16]: 25.45% 2-phenyl-4-(trimethylsilyl) furan; 11.26% 9,17-octadecadienal; 10.92% curlone; 10.31% α-curcumene; 8.1% cis,cis,cis-7,10,13-hexadecatrienal; 7.94% tetradecanal; 7.92% β-sesquiphellandrene; 2.46% zingiberene; 1.79% β-bisabolene; 1.67% 7-methoxymethyl-2,7-dimethylcyclohepta-1,3,5-triene; 0.86% caryophyllene oxide; 0.85% 5-fluoro-1,1,3,3-tetramethyl-1,3-dihydroisobenzofuran; 0.79% thymol; 0.74% linoleic acid; 0.66% p-cymene; 0.66% α–tumerone; 0.61% trans-caryophyllene; 0.53% palmitic acid; 0.47% benzaldehyde.

### 3.3. Growth Performance

In the first experiment, the initial body weight (BW) of the birds was not different (*p* > 0.05) among the dietary treatments (Table 3). Body weight gain (BWG) was found to be significantly highest in the control group during the starter phase (from 1 to 21 days) (*p* < 0.001) and the overall phase (from 1 to 35 days) (*p* = 0.014), showing a linear decreasing effect. However, BWG was not affected during the finisher phase. Feed intake (FI) was higher (*p* = 0.031) in the control group only during the starter phase than in the other groups, which showed a linear effect. Similarly, the feed conversion ratio (FCR) was significantly better in the control group only during the starter phase (*p* = 0.047), which showed a linear effect but was not different during the finisher and overall phases compared to other treatments.

In the second experiment, the initial BW of the birds was not different (*p* > 0.05) among the dietary treatments (Table 4). During the starter (from 1 to 21 days) and overall phase (from 1 to 42 days), the BWG decreased linearly with increasing FS levels (*p* < 0.001 and *p* = 0.001, respectively), with the highest BWG in the control group. On the contrary, the BWG showed no difference (*p* > 0.05) in the finisher phase (from 21 to 42 days of age). FI was significantly higher in the control group during the starter and overall phase (*p* < 0.001 and *p* = 0.003, respectively), showing a linear decreasing effect. However, FI was not significantly different among experimental groups (*p* = 0.283) during the finisher phase. The FCR showed a linear response to increasing FS levels, but only during the starter phase (*p* < 0.001), with better FCR in the control and FS5 groups than FS10. The FCR was similar among dietary treatments (*p* > 0.05) during the finisher and overall phase.

### 3.4. Hematological Parameters

The hematological parameters of male broilers fed with different levels of FS are presented in Table 5. The white blood cell (WBC) count showed a linear increase (*p* < 0.05) with the inclusion of 10 g FS compared to the control and FS5 groups. However, the hematocrit percentage decreased linearly with increasing FS levels (*p* = 0.004). Heterophil and lymphocyte counts showed linear and quadratic responses to increasing FS levels (*p* < 0.05), which peaked in the 10 g FS treatment. Simultaneously, the total protein levels in the blood tended (*p* = 0.058) to decrease with the increasing level of inclusion of FS.

### 3.5. Serum Biochemistry

The influence of different levels of FS on the serum biochemistry of broilers at 42 days of age is presented in Table 6. The inclusion of FS in the broiler diet showed no difference (*p* > 0.05) in glucose, total protein, albumin, globulin, albumin: globulin ratio, GGT, BUN, sodium, potassium, and chloride compared to the control group. However, the total bilirubin levels decreased linearly (*p* = 0.001) with the inclusion of FS, with the highest value in the control group compared to others. The albumin and albumin to globulin ratio tended to decrease (*p* = 0.096 and *p* = 0.062, respectively) in the treated groups compared to the control, which showed a quadratic effect.

### 3.6. Intestinal Histomorphology

The mean values of jejunal and ileal villus height (Vh), villus width (Vw), crypt depth (Cd), and villus height/crypt depth ratio (Vh/Cd) are presented in Table 7. In the jejunum, the Vh and Vh/Cd were significantly decreased with the inclusion of FS (*p* = 0.003 and *p* < 0.001, respectively) compared to the control group. However, the Vw and Cd increased linearly with increasing FS levels (*p* < 0.001), with the highest values in the 10 g FS group compared to other groups.

In the ileum, the Vh was not affected by the dietary inclusion of FS (*p* > 0.05). However, the Vw was linearly increased with increasing levels of FS (*p* < 0.001), with the highest values with the inclusion of 10 g FS compared to others. Moreover, the Cd was also increased (*p* < 0.001) with the FS inclusion compared to the control group. On the contrary, the Vh/Cd was significantly decreased (*p* < 0.001) with the inclusion of FS in the diet.

**Figure 1:**
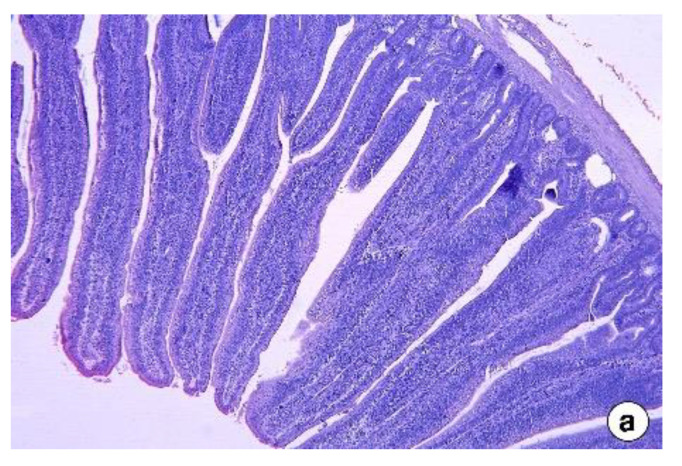

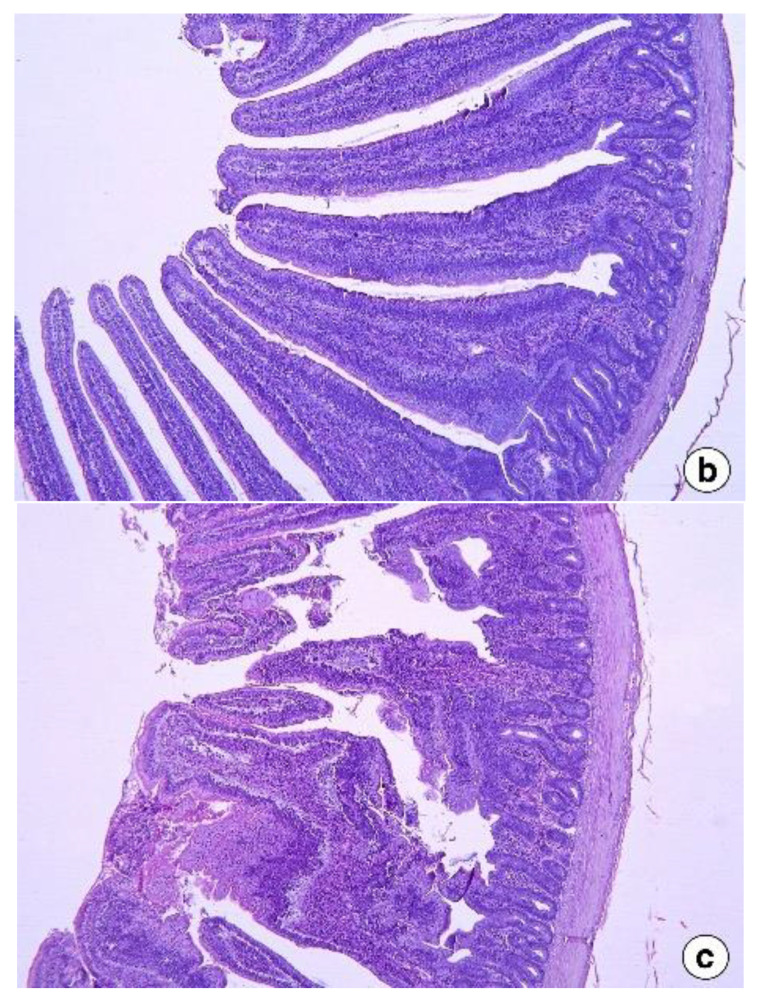
Histomorphology of the jejunum of broilers at 42 days of age from the control group (**a**), FS5 group (**b**), and FS10 group (**c**) (H & E stain, ×50 magnification).

**Figure 2:**
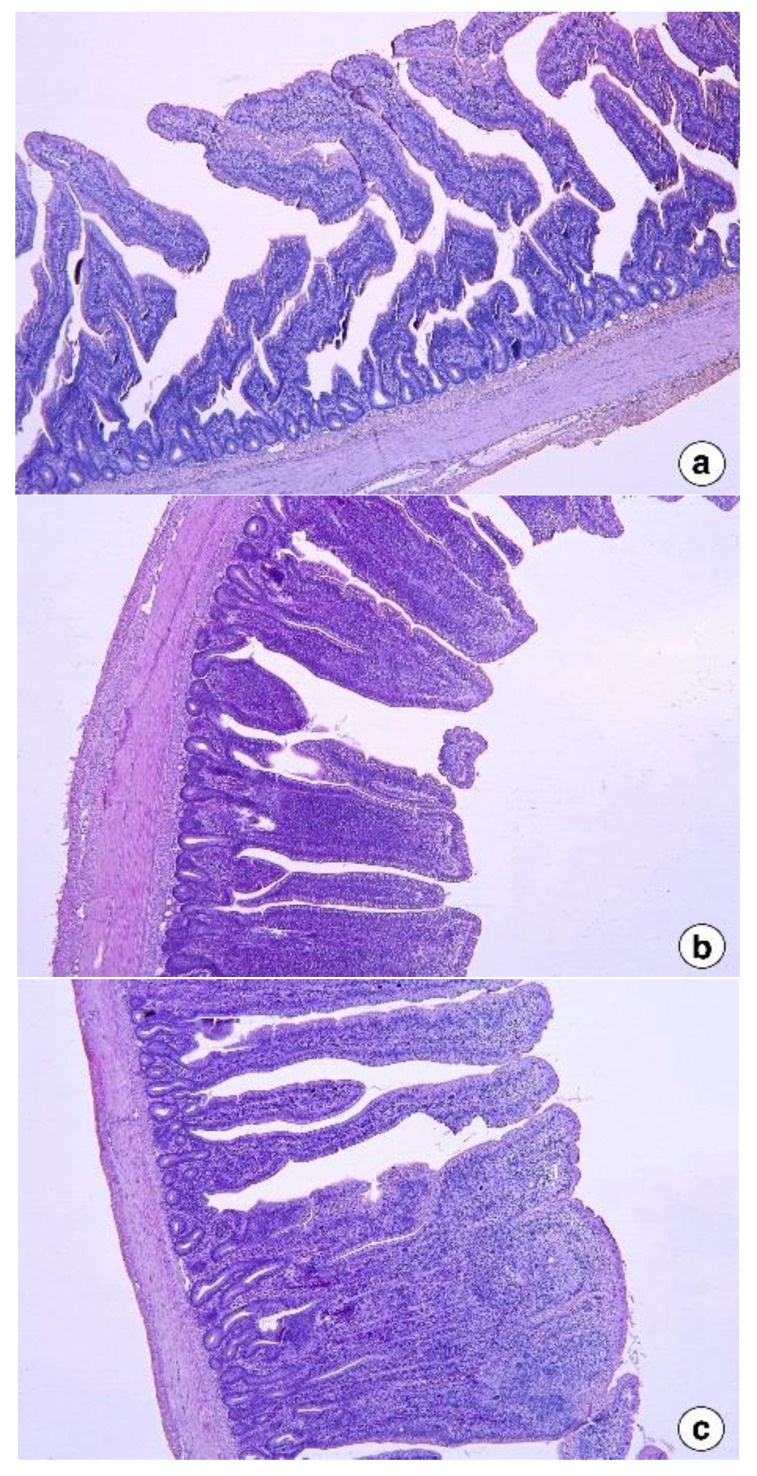
Histomorphology of the ileum of broilers at 42 days of age from the control group (**a**), FS5 group (**b**), and FS10 group (**c**) (H & E stain, ×50 magnification).

The effect of dietary inclusion of FS on the histo-morphological changes in the jejunum and ileum of broilers is presented in Figure 1 and Figure 2, respectively. The jejunum and ileum showed inflammation with the inclusion of 5 g FS, which became more pronounced and prevalent with 10 g FS inclusion in the diet. Damage to the architecture of the jejunum was observed with the inclusion of 10 g FSP.

## 4. Discussion

This study examined the effect of fenugreek seeds on the growth performance, hematological parameters, and intestinal histomorphology of broiler chickens. Two experiments were conducted independently. The first experiment evaluated the effect of four different levels of fenugreek seed powder (0 g, 2.5 g, 5 g, and 10 g/kg of diet) on the growth performance of straight-run broilers. The second experiment examined the effect of three different levels of fenugreek seed powder (0 g, 5 g, and 10 g/kg of diet) on the growth performance, hematological parameter, and intestinal histomorphology of male broilers.

The first experiment showed a linear decrease (*p* < 0.05) in BWG and FI, whereas a linear increase (*p* < 0.05) in FCR with increasing levels of fenugreek seeds during the starter phase in straight-run broilers. However, no effects were observed on the BWG, FI, and FCR during the finisher phase. BWG was also linearly decreased (*p* < 0.05) in the overall phase with the increasing levels of FS. In line with the first experiment, Laudadio et al. (2020) [20] reported the reduction in overall growth performance of broilers with the inclusion of 5 g/kg and 10 g/kg of fenugreek seeds in the broiler diet. Al-Homidan et al. (2020) [23] also reported the dose-dependent reduction of body weight in broilers with the inclusion of 5 g, 15 g, and 25 g/kg FS of fenugreek seeds. In contrast, other studies reported improvement in the growth performance of broilers with the inclusion of 3 g/kg or lower levels of fenugreek seed powder in the diet [24,25]. The decrease in the BWG might be driven by the reduced feed intake during the starter period. The reduced overall performance might be associated with the reduced palatability and lower feed utilization of the diets during the adaptation period of broiler chicken. Fenugreek seeds contain two main constituents that cause a strong odor and bitter taste: volatile oils and alkaloids [15], which might have reduced the palatability of feed. Future research should investigate the organoleptic properties of FS and the intestinal morphometric changes during the starter phases to unfold the taste preferences and histological changes that might be associated with the reduction in feed intake and feed utilization. However, the body weight gain and feed intake were not affected during the finisher phase. Duru et al. (2013) [26] also reported the unchanged body weight gain and feed intake from 22 to 42 days of age, including 5 g, 10 g, 20 g, and 40 g/kg fenugreek [26]. Therefore, it appears that younger broilers are more sensitive to fenugreek, possibly due to being more responsive to factors affecting palatability and/or more susceptible to factors affecting their gastrointestinal system. Birds fed the control diet exhibited the best feed conversion ratio in this experiment. The feed conversion ratio was significantly increased (*p* < 0.05) with the increasing levels of fenugreek seeds and recorded maximum value in 10 g/kg fenugreek seeds inclusion. The increase in feed conversion ratio along with the reduction of body weight gain might be due to poor digestion and absorption of nutrients in the diet. It is also possible that higher fenugreek seed levels may amplify adverse effects on growth performance due to higher doses of bioactive compounds such as alkaloids and steroidal sapogenins delivered by fenugreek seeds.

The second experiment also showed a linear decrease (*p* < 0.05) in BWG and FI, whereas a linear increase (*p* < 0.05) in FCR with increasing levels of fenugreek seeds (0 g, 5 g, and 10 g/kg FS) was noted during the starter phase in male broilers. However, no effects (*p* > 0.05) were observed on the BWG, FI, and FCR during the finisher phase. BWG and FI were linearly decreased (*p* < 0.05) in the overall phase with the increasing levels of FS. These results were similar to the results obtained in the first experiment with straight-run broilers.

WBC count, heterophil count, and lymphocyte count were linearly increased (*p* < 0.05) with increasing levels of FS in the diet. It might be possible that inflammation and damage to the jejunum that was characterized by infiltrations of WBC may have led to an increase in the WBC count, heterophil count, and lymphocyte counts. In addition, there was a decrease in the hematocrit percentage compared to the control and 5 g FS inclusion. However, the values lie within the normal hematological range of broiler chickens. Alterations in the hematological count might result from inhibition of hematopoietic regulatory elements in the bone marrow with the high inclusion of FS in the diet [27].

Dietary fenugreek seeds inclusion did not influence (*p* > 0.05) the serum biochemical parameters of the broilers except for total bilirubin in experiment 2. The total bilirubin levels showed a linear decrease (*p* < 0.05) with increasing levels of fenugreek seeds in the diet. Qureshi et al. (2017) [28] also reported a decrease in total bilirubin with the inclusion of fenugreek in the broiler diet. Lower bilirubin levels in the blood serum indicate no negative effects on liver functions [29].

The intestinal histomorphology studies in the second experiment revealed that the jejunum and ileum showed adverse responses to dietary fenugreek seeds. This included shorter villi and deeper crypts that could have reduced the nutrient digestion and absorption, culminating in reduced body weight gain in FS added groups in the present study compared to control. The small intestine morphology, especially the villi and crypt, plays a vital role in nutrient digestion and assimilation in animals [30]. Long villi and more shallow crypts are important features of gut morphological development in any animal. Longer villi are associated with a larger total luminal absorptive area, leading to better digestive enzyme function and better nutrient transport [31]. Simultaneously, shallower crypts represent a longer turnover time for the renewal of the villus [32], resulting in reduced energy expenditure, and as a result, energy can be channeled to other tissues [33]. The presence of toxins might be associated with alterations in intestinal architecture, such as shorter villi and deeper crypts, resulting in inadequate nutrient absorption, increased gastrointestinal secretion, and reduced growth performance [27,34]. Therefore, the altered intestinal morphology observed in the broiler chickens fed with 5 g and 10 g fenugreek seeds could be related to the toxic effects of high levels of saponins present in the higher inclusion levels of fenugreek seeds. In fact, in the 10 g/kg inclusion of fenugreek, there were instances of damage to the jejunum. Further studies are warranted to analyze the components of fenugreek seeds, such as volatile oils and alkaloids, that affect the intake of the diets in broiler chickens and affect intestinal morphology, especially during the starter phase.

## 5. Conclusions

The results indicate that the dietary inclusion of FS at 2.5 g, 5 g, and 10 g/kg of diet linearly reduced the growth performance, increased the WBC counts, and negatively affected the intestinal morphology of broiler chickens. In particular, growth performance was only reduced during the starter phase with no effects during the finisher phase. Our study concludes that the inclusion of higher levels of FS negatively affects the growth performance and intestinal morphology of broilers. It suggests that the effect of FS on poultry varies depending on the inclusion level and growth phase, with the recovery or beneficial effects occurring during the finisher period.

## Figures and Tables

**Table 1:** Ingredient composition (% as fed) of the experimental basal diet.

Ingredient (%)	Feeding Phase	
	Starter (1–21 Days)	Finisher (21–42 Days)
Corn	59.37	66.47
Soybean meal	32.85	25.25
Pro-Plus ^1^	2.5	0
Meat and bone meal, 50%	0	2.5
Poultry oil	2.01	3.14
Sodium chloride	0.38	0.31
Sodium bicarbonate	0	0.05
Limestone	0.8	0.7
Dicalcium phosphate	1.13	0.85
Vitamin premix ^2^	0.1	0.1
Mineral premix ^3^	0.1	0.1
Choline chloride	0.1	0.1
Selenium PMX 0.06%	0.02	0.02
Santoquin	0.02	0.02
L-Lys HCL	0.17	0.1
DL- Met	0.3	0.21
L-Thr	0.11	0.05
Copper chloride	0.02	0
Xylanase	0	0.01
Phytase	0.02	0.02
Total	100	100

^1^ Pro-Plus is an animal by-product blend, with a CP content of 60% (H. J. Baker & Bros. Inc., Little Rock, AR, USA). ^2^ Vitamin premix (provided the following per kilogram of diet): vitamin A (trans retinyl acetate), 3600 IU; vitamin D_3_ (cholecalciferol), 800 IU; vitamin E (dl-α-tocopheryl acetate), 7.2 mg; vitamin K_3_, 1.6 mg; thiamine, 0.72 mg; riboflavin, 3.3 mg; niacin, 0.4 mg; pyridoxin, 1.2 mg; cobalamine, 0.6 mg; folic acid, 0.5 mg; choline chloride, 200 mg. ^3^ Mineral premix (provided the following per kilogram of diet): Mn (from MnSO_4_·H_2_O), 40 mg; Zn (from ZnO), 40 mg; Fe (from FeSO_4_·7H_2_O), 20 mg; Cu (fromCuSO_4_·5H_2_O), 4 mg; I (from Ca(IO_3_)_2_·H_2_O), 0.64 mg; Se (from sodium selenite), 0.08 mg.

**Table 2:** Analyzed composition (% as dry matter) of the experimental basal diet.

Analyzed Composition (%) ^1^	Starter Basal Diet	Finisher Basal Diet
Dry matter	92.3	92.3
Crude protein	23.1	18.9
Ether extract	4.68	6.5
Neutral detergent fiber	12.3	10.9
Acid detergent fiber	6.2	6.3
Phosphorus	0.71	0.69
Calcium	1.03	0.98
Potassium	1	0.87
Magnesium	0.17	0.15
Sodium	0.229	0.291
Sulfur	0.30	0.22
Iron (ppm)	116	119
Manganese (Mn) ppm	122	211
Zinc (Zn) ppm	157	191
Copper (Cu) ppm	180	43.2

^1^ Values are presented in percentage unless stated.

**Table 3:** Effect of the dietary fenugreek seed (FS) inclusion on the growth performance of straight-run broiler chickens in Experiment 1 ^1^.

	Dietary Treatments ^2^					*p*-Value ^4^	
Parameters ^3^	C	FS2.5	FS5	FS10	SEM	Linear	Quadratic
Initial BW (g)	39	38	38	38	0.19	0.29	0.277
Starter period							
BWG (g)	944 ^a^	824 ^bc^	860 ^b^	771 ^c^	18.00	<0.001	0.389
FI (g)	1172 ^a^	1107 ^ab^	1097 ^ab^	1070 ^b^	16.13	0.031	0.523
FCR (g/g)	1.24 ^b^	1.35 ^ab^	1.27 ^ab^	1.39 ^a^	0.02	0.047	0.909
Finisher period							
BWG (g)	1204	1098	1169	1145	17.72	0.480	0.231
FI (g)	2020	1947	2066	1929	24.54	0.448	0.489
FCR (g/g)	1.68	1.78	1.77	1.68	0.03	0.992	0.094
Overall performance							
BWG (g)	2148 ^a^	1922 ^b^	2029 ^ab^	1917 ^b^	31.95	0.014	0.24
FI (g)	3192	3054	3163	2999	33.78	0.106	0.835
FCR (g/g)	1.49	1.59	1.56	1.56	0.02	0.297	0.235

Means not sharing a common letter (a–c) in a row are significantly different (*p* < 0.05). ^1^ Each mean represents four replicates with ten birds per replicate (n = 40 per treatment). ^2^ Four dietary treatments; C = Control; FS2.5 = 2.5 g/kg inclusion level of fenugreek seed powder; FS5 = 5 g/kg inclusion level of fenugreek seed powder; FS10 = 10 g/kg inclusion level of fenugreek seed powder. ^3^ BWG—bodyweight gain; FI—feed intake; FCR—feed conversion ratio. ^4^ Statistical significance: *p* < 0.05.

**Table 4:** Effect of the dietary fenugreek seed (FS) inclusion on the growth performance of male broiler chickens in Experiment 2 ^1^.

	Dietary Treatments ^2^				*p*-Value ^4^	
Parameters ^3^	C	FS5	FS10	SEM	Linear	Quadratic
Initial BW (g)	43	43	43	0.30	0.291	1
Starter period						
BWG (g)	983 ^a^	872 ^b^	730 ^c^	26.66	<0.001	0.452
FI (g)	1274 ^a^	1163 ^b^	1050 ^c^	23.96	<0.001	0.976
FCR (g/g)	1.30 ^b^	1.34 ^b^	1.44 ^a^	0.02	<0.001	0.209
Finisher period						
BWG (g)	1848	1841	1811	24.94	0.566	0.844
FI (g)	3264	3247	3159	37.87	0.283	0.671
FCR (g/g)	1.77	1.77	1.75	0.02	0.642	0.788
Overall performance						
BWG (g)	2831 ^a^	2713 ^ab^	2541 ^b^	39.77	0.001	0.67
FI (g)	4538 ^a^	4409 ^ab^	4209 ^b^	48.41	0.003	0.66
FCR (g/g)	1.60	1.63	1.66	0.01	0.061	0.848

Means not sharing a common letter (a–c) in a row are significantly different (*p* < 0.05). ^1^ Each mean represents six replicates with eight birds per replicate (n = 48 per treatment). ^2^ Three dietary treatments; C = Control; FS5 = 5 g/kg inclusion level of fenugreek seed powder; FS10 = 10 g/kg inclusion level of fenugreek seed powder. ^3^ BWG—bodyweight gain; FI—feed intake; FCR—feed conversion ratio. ^4^ Statistical significance: *p* < 0.05.

**Table 5:** Effect of the dietary fenugreek seed (FS) inclusion on the hematological parameters of the broiler chickens ^1^.

Parameters	Dietary Treatments ^2^				*p*-Value ^3^	
	C	FS5	FS10	SEM	Linear	Quadratic
WBC count (10^3^ cells/µL) ^4^	15.83 ^b^	14.72 ^b^	23.41 ^a^	1.12	0.002	0.020
Hemoglobin (g/dL)	10.59	10.12	10.01	0.13	0.065	0.509
Hematocrit (%)	29.08 ^a^	27.91 ^ab^	26.58 ^b^	0.37	0.004	0.916
Total protein (g/dL)	2.97	2.90	2.76	0.05	0.058	0.694
Heterophil count (10^3^ cells/µL)	5.69 ^b^	4.99 ^b^	8.28 ^a^	0.41	0.003	0.010
Lymphocyte count (10^3^ cells/µL)	8.76 ^b^	8.05 ^b^	12.59 ^a^	0.65	0.009	0.041
Monocyte count (/µL)	63.92	73.64	152.42	27.04	0.184	0.556
Basophil count (10^3^ cells/µL)	1.28	1.02	2.09	0.19	0.069	0.093

Means not sharing a common letter (a–b) in a row are significantly different (*p* < 0.05). ^1^ Each mean represents six replicates with two birds per replicate (n = 12/treatment). ^2^ Three dietary treatments; C = Control; FS5 = 5 g/kg inclusion level of fenugreek seed powder; FS10 = 10 g/kg inclusion level of fenugreek seed powder. ^3^ Statistical significance: *p* < 0.05. ^4^ WBC—white blood cells.

**Table 6:** Effect of the dietary inclusion of different levels of fenugreek seed (FS) on the serum biochemistry of the broiler chickens ^1^.

	Dietary Treatments ^2^				*p*-Value ^3^	
Parameters	Control	FS5	FS10	SEM	Linear	Quadratic
Glucose (mg/dL)	216	219	222	2.89	0.424	0.968
Total protein (g/dL)	2.63	2.53	2.63	0.04	1.000	0.235
Albumin (g/dL)	0.73	0.66	0.68	0.01	0.186	0.096
Globulin (g/dL)	1.90	1.88	1.95	0.03	0.520	0.458
Albumin/globulin ratio	0.38	0.33	0.36	0.01	0.408	0.062
GGT (IU/L) ^4^	29	27	29	0.73	0.782	0.206
BUN (mg/dL) ^5^	2.33	2.33	2.25	0.09	0.707	0.828
Sodium (mEq/L)	152	148	148	1.16	0.134	0.500
Potassium (mEq/L)	4.81	4.90	5.34	0.16	0.620	0.620
Chloride (mEq/L)	2.91	7.45	3.91	0.85	0.367	0.511
Total bilirubin (mg/dL)	0.38 ^a^	0.29 ^b^	0.27 ^b^	0.01	0.001	0.270

Means not sharing a common letter (a–b) in a row are significantly different (*p* < 0.05). ^1^ Each mean represents six replicates with two birds per replicate (n = 12/treatment). ^2^ Three dietary treatments; C = Control; FS5 = 5 g/kg inclusion level of fenugreek seed powder; FS10 = 10 g/kg inclusion level of Fenugreek seed powder. ^3^ Statistical significance: *p* < 0.05. ^4^ GGT—Gamma-glutamyl transferase; ^5^ BUN—blood urea nitrogen.

**Table 7:** Effect of the dietary FS inclusion on the histomorphology of jejunum and ileum of broiler chickens ^1^.

	Treatments ^2^				*p*-Value ^4^	
Parameters ^3^	C	FS5	FS10	SEM	Linear	Quadratic
Jejunum						
Vh (μm)	1650 ^a^	1438 ^b^	1416 ^b^	33.38	0.003	0.144
Vw (μm)	138 ^c^	183 ^b^	215 ^a^	6.54	<0.001	0.494
Cd (μm)	154 ^c^	204 ^b^	236 ^a^	7.31	<0.001	0.39
Vh/Cd (μm/μm)	10.79 ^a^	7.29 ^b^	6.19 ^b^	0.38	<0.001	0.024
Ileum						
Vh (μm)	712	686	672	18.71	0.384	0.891
Vw (μm)	107 ^c^	146 ^b^	188 ^a^	17.03	<0.001	0.93
Cd (μm)	119 ^b^	158 ^a^	184 ^a^	6.77	<0.001	0.601
Vh/Cd (μm/μm)	5.98 ^a^	4.66 ^b^	3.81 ^b^	0.22	<0.001	0.537

Means not sharing a common letter (a–c) in a row are significantly different (*p* < 0.05). ^1^ Each mean represents six replicates with two birds per replicate (n = 12/treatment). ^2^ Three dietary treatments; C = Control; FS5 = 5 g/kg inclusion level of fenugreek seed powder; FS10 = 10 g/kg inclusion level of Fenugreek seed powder. ^3^ Vh—villus height; Vw—villus width; Cd—crypt depth; Vh/Cd—villus height to crypt depth ratio. ^4^ Statistical significance: *p* < 0.05.

## Data Availability

Not applicable.
